# The *Meta Salud Diabetes* Implementation Study: Qualitative Methods to Assess Integration of a Health Promotion Intervention Into Primary Care to Reduce CVD Risk Among an Underserved Population With Diabetes in Sonora, Mexico

**DOI:** 10.3389/fpubh.2019.00347

**Published:** 2019-11-15

**Authors:** Maia Ingram, Catalina A. Denman, Elsa Cornejo-Vucovich, Maria del Carmen Castro-Vasquez, Benjamin Aceves, Abraham Garcia Ocejo, Jill Guernsey de Zapien, Cecilia Rosales

**Affiliations:** ^1^Department of Health Promotion Sciences, Mel and Enid Zuckerman College of Public Health University of Arizona, Tucson, AZ, United States; ^2^Center for Health and Society Studies, El Colegio de Sonora, Hermosillo, Mexico; ^3^Department of Epidemiology, Mel and Enid Zuckerman College of Public Health University of Arizona, Tucson, AZ, United States; ^4^Division of Public Health Practice and Translational Research, Mel and Enid Zuckerman College of Public Health University of Arizona, Tucson, AZ, United States

**Keywords:** implementation science, qualitative methods, health promotion, Mexico, primary care, diabetes, cardiovascular disease

## Abstract

**Background:** Within health promotion research, there is a need to assess strategies for integration and scale up in primary care settings. Hybrid interventions that combine clinical effectiveness trials with implementation studies can elicit important contextual information on facilitators and barriers to integration within a health care system. This article describes lessons learned in developing and implementing a qualitative study of a cluster-randomized controlled trial (RCT) to reduce cardiovascular disease (CVD) among people with diabetes in Sonora, Mexico, 2015–2019.

**Methods:**The research team worked cooperatively with health center personnel from 12 Centers that implemented the intervention. The study used observations, stakeholder meetings, case studies, staff interviews and decision maker interviews to explore issues such as staff capacity, authority, workflow, space, and conflicting priorities, as well as patients' response to the program within the clinical context and their immediate social environments. Applying a multi-layered contextual framework, two members of the research team coded an initial sample of the data to establish inclusion criteria for each contextual factor. The full team finalized definitions and identified sub nodes for the final codebook.

**Results:** Characteristics of management, staffing, and the local environment were identified as essential to integration and eventual adoption and scale up across the health system. Issues included absence of standardized training and capacity building in chronic disease and health promotion, inadequate medical supplies, a need for program monitoring and feedback, and lack of interdisciplinary support for center staff. Lack of institutional support stemming from a curative vs. preventive approach to care was a barrier for health promotion efforts. Evolving analysis, interpretation, and discussion resulted in modifications of flexible aspects of the intervention to realities of the health center environment.

**Conclusion:** This study illustrates that a robust and comprehensive qualitative study of contextual factors across a social ecological spectrum is critical to elucidating factors that will promote future adoption and scale up of health promotion programs in primary care. Application of conceptual frameworks and health behavior theory facilitates identification of facilitators and barriers across contexts.

**Trial registration:** www.ClinicalTrials.gov, identifier: NCT02804698 Registered on June 17, 2016.

## Introduction

Rising rates of non-communicable diseases pose a major threat to the health of Mexicans and are contributing to a slowing in increased life expectancy (1, 2) Type 2 diabetes is the second highest cause of death for both men and women in Mexico (3), and the combined threat of diabetes and cardiovascular disease (CVD) accounts for one-third of deaths among Mexican adults (1). Three of four adults with diabetes in Mexico have blood pressure above recommended levels, and half have hypertension (2), placing them at extreme risk for CVD. Mexico has declared a national emergency in the face of the epidemic (4), with quality of care at the center of combatting the disease (5). In fact, only 24% of people with diabetes in Mexico have diabetes-related outcomes that are considered under control (1). In addition to the timely detection of the disease, the quality of primary care, including disease monitoring and access to medications, present major challenges. There is an urgent need to evaluate and implement prevention and control programs for non-communicable diseases in Mexico (2).

There is ample evidence that health promotion interventions have a positive impact on health behaviors and contribute to prevention and control of non-communicable diseases (6, 7). These documented outcomes often rely upon conditions created in the context of clinical trials, which invariably include resources to ensure staffing, training, recruitment, incentives, and even adequate facilities and materials to implement the intervention. Relatively well-funded clinical trials fail to account for the training and staffing challenges, dueling priorities, and scarcity of resources inherent in real world global settings, creating significant gaps between the effectiveness of a health promotion intervention and the capacity for health systems to deliver the intervention to the target population in a sustainable way. Implementation science helps to bridge this gap by simultaneously exploring contextual factors influencing implementation and identifying methods to integrate evidence-based programs and practices successfully into the health care delivery system (8–10). Given the complexity of health care systems and the challenges of translating research into clinical practice (11), there is a need for flexible and tailored research methods in the study of context.

Hybrid interventions combine clinical effectiveness trials with implementation studies (9). In this scenario, researchers work with practitioners to carry out randomized control trials (RCTs) of interventions within the context of a particular health system. These types of studies are particularly useful in informing and facilitating eventual adoption and scale up of the intervention, because they engage stakeholders and decision makers in the potential value of the intervention at the outset, while concurrently identifying issues that need to be addressed to make broad implementation of the intervention achievable (12). A major contribution of these studies is that they move beyond studying factors related to trial participants, to include the professionals ultimately responsible for administering the program, as well as factors of the organization and health system responsible for sustaining the intervention (13, 14). Latin American countries can benefit from greater utilization of complex studies because it has proven difficult to adapt and integrate evidence-based programs developed elsewhere into existing health systems (15–17). However, we found no published studies of this nature from Mexico.

Qualitative methods contribute to greater understanding of the complexity of contextual factors influencing intervention implementation (18), and can be employed across the stages of an RCT to improve study design, inform study results and identify more flexible aspects of an intervention that can be adapted to different contexts (19, 20). In this article, we describe lessons learned from the evolving process of conducting a qualitative implementation study alongside a cluster RCT of *Meta Salud Diabetes* (MSD) (Diabetes Health Goal), a CVD prevention curriculum targeting uninsured patients with diabetes served by the *Secretar*í*a de Salud*, or Ministry of Health, in Sonora, Mexico. By conducting our study within the context of a cluster RCT, our objective was not only to document factors that impact implementation, but also to demonstrate the utility of iterative and responsive qualitative methods in identifying contextual factors that will inform future efforts to translate MSD and other intervention research into practice based settings (21).

## Materials and Methods

The cooperative relationship that the research team developed with the Ministry of Health in prior projects was crucial to our ability to conduct the current study. By working directly with state and local health personnel, we sought to ensure that we identified complexities related to implementation within specific health centers and the healthcare system, while generating generalizable information from an implementation process that could be applicable to other settings. In this project, we were able to build upon the extensive experience of the lead researchers in Sonora and Arizona working directly with the Ministry of Health on health promotion research (22, 23). In addition, we hired two well-respected physicians from the Ministry of Health to assist in the development of study protocols with the 22 centers involved in the study.

### Mexico Health Care Delivery System

The Mexico Ministry of Health has centralized health insurance programs that provide coverage to most adults through state and national networks of hospitals and clinics. *Seguro Popular*, the national health insurance program initiated in 2004, serves the most vulnerable population (56%) of Mexico through a network of health centers and hospitals *Seguro Popular*, which is financially independent of the Ministry of Health, provides a specific service package at minimal or zero cost that includes diabetes and other catastrophic diseases. However, because the program covers explicit health care interventions rather than the disease itself, procedures to address the complications of diabetes may not be covered, creating a health and financial burden for Mexico's vulnerable populations (24) (As of this writing, *Seguro Popular* has been suspended by the federal government and will be substituted by a different scheme.) Self-help groups, known as Grupos de Ayuda Mutua (GAM), constitute a strategy utilized by the Mexico Ministry of Health since 1999 to address secondary prevention among their uninsured patients with diabetes (25). National GAM guidelines outline a general approach that includes weekly meetings in which members monitor their glucose, receive health information, and cultivate a supportive group environment for self-management. In addition to providing an infrastructure of self-management education and monitoring, the GAM guidelines encourage cooperation and reciprocity among health center personnel, specifically in terms of sharing resources, skills and services for the benefit of diabetes patients. Health centers register patient data with the Health Ministry to gain accreditation and recognition for excellence (26). There are currently more than 10,000 of these groups nationally, which cover over 100,000 patients (26). As a strategy promoted nationally, the extent to which GAMs are utilized within clinical care and the quality of the program varies from state to state depending on prioritization, funding, trained personnel, and adequate supervision (27).

The project posited that the infrastructure created through the GAM within the government-run health centers (*Centros de Salud*) would represent an ideal system for future scale up of an evidence-based intervention for CVD risk management in people with diabetes. Many populations in Mexico are eligible and receive care at these clinics and Mexico's centralized health care system will greatly facilitate integration across a large number of clinics. However, health care delivery systems are complex and the acceptance of any new intervention will be influenced by multiple factors. Issues of importance to scale up include the identification of and fidelity to components of the intervention that are most vital to health outcomes, as well as ways in which the intervention is integrated into the overall delivery of health services to maximize effectiveness (28). It is therefore essential to study factors and barriers related to the implementation of an evidence-based practice or program.

With assistance from the Ministry of Health in Sonora, the research team recruited 22 (22) health centers that had an active GAM and in which the directors agreed to be randomized to intervention or control arms using geographical stratification. The RCT is described in detail in Sabo et al. using the Consolidated Standards for Reporting Trials framework (29). The implementation study included the 12 centers randomized into the intervention arm. Each Center Director identified two to three staff who were responsible for facilitating and/or supporting the GAM to participate in the MSD training, which was provided by the research team. GAM facilitators included nurses, health promoters and physicians. Research staff provided a 2-day16-h training prior to implementation with an 8-h booster half way through the 13-week intervention, following session 6. In addition to content, the trainings included critical reflection on preventive vs. curative care, and focused on the use of participatory and dynamic communication techniques. The booster session was developed in response to implementation observations made by the research team and began with a general sharing and discussion of the experience of facilitating the MSD intervention. Training focused on strategies to conduct physical activity sessions in limited space with participants who may have physical limitations, and a review of specific content areas in the final six sessions, such as cholesterol and fat, medications, and emotional health. Each Center received an MSD facilitation handbook and workbooks for all participants.

### Meta Salud Diabetes (MSD)

As an evidence-based intervention, the development of the MSD curriculum has antecedents in a binational collaboration between *El Colegio de Sonora* in Hermosillo, Mexico and the University of Arizona in Tucson, Arizona. The partnership initially adapted a U.S.-based health promotion program *Pasos Adelante* (Steps Forward) (30, 31), which the Mexico partner subsequently re-conceptualized as *Meta Salud* (Goal Health). *Meta Salud* sought to better reflect the sociocultural and institutional characteristics of Northern Mexico, and bolster the participatory practices of the facilitators to promote the agency and empowerment of participants (23). In this third iteration*, Meta Salud Diabetes* (MSD) is a secondary prevention curriculum addressing CVD risk among people with diabetes. As a health promotion curriculum designed for primary care settings, MSD was tailored for the health expertise of health center personnel responsible for facilitating the GAMs, and organized to correspond to the existing GAM structure by conforming to the time frame, facilities and personnel, as well as accommodating regular glucose monitoring (29). In addition to incorporating diabetes information and self-management practices, a central characteristic of MSD is to encourage health staff to move beyond primarily curative care toward a holistic and health promoting approach to diabetes management. For intervention participants, MSD incorporates socioecological and gender perspectives, placing emphasis on the role of the family and immediate social circle in diabetes self-management, as well as an analysis of how community resources can be leveraged to promote health. Group discussions foster goal setting and participants learn skills such as reading food labels and making cheap and easy healthy meals to increase their sense of self- efficacy, and make plans to identify and keep potential environmental factors from disrupting their goals. Considering social context, the curriculum encourages participants to think about personal and collective agency and their right to quality health services.

### Conceptual Framework

**Figure 1:**
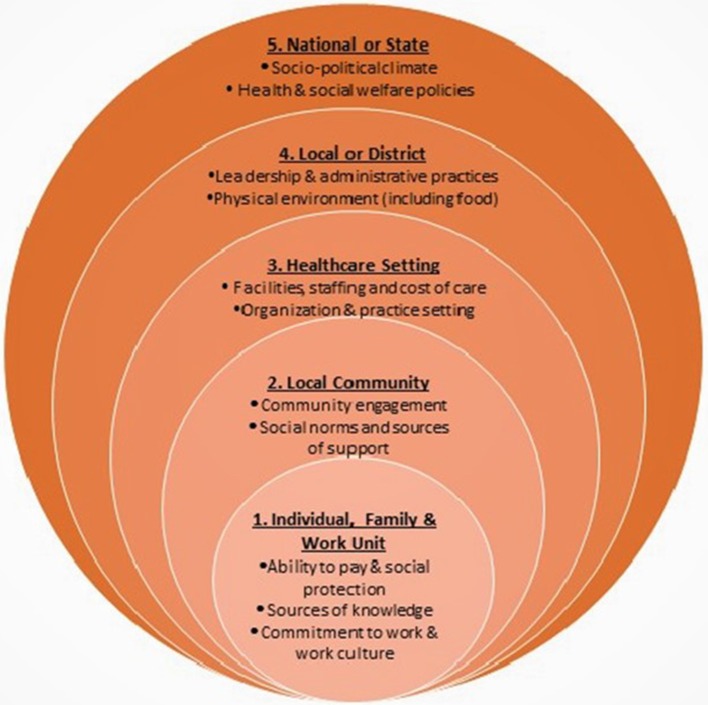
Multi-layered framework for measuring context in clinical settings based on Taplin et al. (32).

In designing the study, the use of theoretical and conceptual frameworks was essential to our ability to identify contextual factors that could be relevant to integration of MSD into primary care. The multi-layered context framework shown in Figure 1 (32, 33) is similar to the social ecological model in that it considers the interactional layers of influence within a system (34). The elements at each level of influence in the figure were drawn from the Context Assessment for Community Health (COACH) tool (35), and capture the complexity of contextual factors influencing chronic disease prevention. In addition to the contextual model, we applied theoretical constructs that helped us to posit specific dynamics that might contribute to the effectiveness of the intervention within the clinical setting. We used the theory of salutogenesis to guide identification of implementation outcomes regarding ways in which how health personnel encourage participants to identify and build upon current practices that support the health and well-being of individuals with diabetes, rather than focusing on the state of their disease (36, 37). GAM facilitators, for example, are responsible for encouraging group interaction aimed at capitalizing on existing knowledge and encouraging critical self-reflection that is respectful of cultural mores and values. In investigating factors relevant to the context of the health centers we applied Normalization Process Theory (NPT) to explore staff interest, capacity and resources to implement the MSD intervention within the existing GAM structure (38, 39). Additionally, a systematic review of studies of context in intervention implementation conducted by Blacklock et al. identified a potential influencing factors related to physical space, organizational structure, leadership, and staff training (40). We also sought to explore state and federal health system settings and the economic, political and social factors that create or inhibit opportunities in public health policy (41).

### Qualitative Data Collection Methods

The implementation study included systematic qualitative data collection across the contextual layers before, during and after implementation of the *Meta Salud Diabetes* intervention, with a focus on the health care staff and health care setting. A variety of qualitative methods facilitated in depth inquiry into the feasibility of the intervention within a complex organizational environment that would provide a range of perspectives and be relevant across settings. The study explored issues such as staff capacity, particularly with respect to health promotion and disease prevention, authority, work flow, space, and conflicting priorities such as curative care and adequate medication, and patients' response to the program within the clinical setting and within the context of their immediate social environments. We designed our data collection activities to target each layer of context and developed instruments based on the relevant theoretical constructs. The development of evaluation instruments was iterative in that analysis from each data source helped to define future questions. In most cases, the activities and instruments were also crosscutting across the layers. Table 1 outlines the implementation timeline, corresponding data collection activities across the layers of context and the timeline for the activity. Data collection instruments targeted each layer of context, but also intersected the layers.

**Table 1:** Implementation study activities of Meta Salud Diabetes across layers of context (40).

**Method**	**Layer(s) of context**	**Description**	**Timeline**
Health center evaluations	•Local or district •Health care setting	Staff directories, informal observation, and conversations with health center staff to evaluate interest and ability in intervention	Pre intervention
Health center stakeholder meetings	•Health care setting	Participatory meetings designed to elicit priorities of health center staff and to identify strategies and scenarios for integration of MSD	Pre and post intervention
MSD observation	•Health care setting •Local community •Patient, family, and work unit	Structured observation of 13 MSD sessions to document fidelity, as well as factors related to the participant and staff interactions, group dynamics, and how participants and staff responded to the intervention.	During intervention
MSD facilitator meetings	•Health care setting •Patient, family, and work unit	Structured discussions with facilitators from 11 centers related to MSD training needs and facilitators and barriers to implementation.	Midway and post intervention
Center case studies (4)	•Local or district •Health care setting •Patient, family, and community	Participant focus groups and health center personnel interviews designed to identify facilitators and barriers to MSD implementation and scale up as well as to sustained participant self-management.	Post intervention
Decision-maker interviews	•National, state and international •Health care setting •Patient, family, and community	Semi-structured interviews with Ministry of Health personnel and key informants from other non/governmental agencies to identify facilitators and barriers to scale up of MSD within state and federal health system	Post intervention

#### Health Center Condition Assessment

Prior to initiation of the RCT, research staff visited the 12 health centers to observe the clinical environment and the physical space where GAM facilitators convened the group. We also engaged in informal conversations with center directors and GAM facilitators regarding the availability of human resources and infrastructure to facilitate MSD. We took detailed notes of these conversations, as well as photographs of the physical space.

#### Stakeholder Meetings

Before initiation of the RCT of MSD, the research team held separate stakeholder meetings with the 12 intervention health centers. Health centers were generally responsive to requests for participation of the center director, the clinical director of non-communicable diseases, and health promotion staff, and often, all health center staff participated. The stakeholder meetings were designed to elicit priorities of center staff in addressing diabetes and CVD risk. We also sought to identify strategies and scenarios in which health centers could integrate an intervention such as MSD into the ongoing structure of the GAM. The research team facilitated small group conversations in which they posited questions related to the care of non-communicable diseases and the role of the GAM and of MSD. A second stakeholder meeting was conducted at intervention sites after the program had concluded. The research team took notes and audiotaped the meetings and stakeholders recorded their conversations on worksheets based on discussion prompts.

#### MSD Implementation Observation

Members of the research team provided a liaison to each of the 12 centers randomized to implement the MSD. The team member observed each of the 13 sessions using non-participant observation methodology in which the researcher is in the room during the intervention, but does not take part in activities or offer advice to the facilitator. The observers did not intervene to modify the dynamics, but rather sat in the back or to one side, took notes and only if extraordinary circumstances required it they spoke with the GAM facilitator at the end of the session. They registered observations in detailed, extensive field diaries. The observations focused on: (1) fidelity to the intervention design; (2) other factors related to the facilitation by each of the GAM facilitators; (3) the involvement and participation of GAM participants in the MSD intervention; (4) ways in which information was shared between participants; (5) the social interactions and dynamics of the group; and (6) the level of social support within the GAM and how it was perceived and received. Feedback meetings with MSD facilitators.

After each of the three intervention cycles the research team facilitated feedback meetings with GAM/MSD facilitators and those that provided support to the intervention. Responsibility for facilitating the MSD intervention within the context of the GAM varied across the health centers. While nurses most often took the lead in facilitating the GAM/MSD, they often had the support of other staff, including nurses, community health workers, and interns (Table 2). Research staff developed meeting content based on discussion of needs that facilitators and research liaisons had identified during the intervention. Each meeting was recorded in detailed notes for data analysis.

**Table 2:** Health personnel involved in Meta Salud Diabetes (MSD) Study.

**Health center personnel involved in MSD facilitation (12 centers)**	
**Position (12 centers)**	**Number**
Physician	4
Nurse	14
Nutritionist	1
Social worker	1
Psychologist	1
Intern (psychology, nutrition, nursing)	8
Community health worker	6
**Case study interviews (4 centers)**	
Director	4
Sub Director	1
Physician/Director of chronic disease care	2
Nurse who facilitated MSD	5
Community health worker	3
**Decision-maker interviews (State and Federal level)**	
Federal Program Director	4
Federal Program Sub-Director	3
State Program Director	2
State Program Sub-director	3
Other key informants (academic, NGO)	3

#### Center Case Studies

In addition to the initial visit, the stakeholder meetings and the observations, in four intervention health centers we also conducted focus groups with MSD participants and interviews with health staff including the center director, the GAM facilitators, and providers responsible for chronic disease care. The main purpose of the case study was to identify factors that both facilitated and challenged MSD implementation, and to situate these factors within the broader context of health care delivery in the health center. We selected the four centers following implementation of MSD based on our perception that the case studies would yield rich information related to the implementation questions (42). We selected cases from three distinct regions in the state representing groups with varying strengths and challenges in implementation with respect to factors such as consistent attendance, intervention fidelity, facilitator commitment, and institutional support. With the exception of one center, four staff from the each of the case study health centers participated in interviews (see Supplementary Materials). The health center directors and those responsible for the GAM/MSD intervention participated from each center. In two centers, the physician overseeing chronic disease care participated (Table 2).

#### State and Federal Decision Maker Interviews

To understand broader contexts related to the feasibility and financing/sustaining of implementation, we conducted semi-structured interviews with seven federal and five state health promotion and chronic disease prevention leadership, as well as with academic and international leaders in health promotion (see Supplementary Materials).

### Ethics Approval and Consent to Participate

The study protocol and consent process was approved by the University of Arizona Human Subjects Institutional Review Board. The protocol was also approved by the Research Bioethics Committee at the University of Sonora. Center staff consented to session observation upon recruitment and clinic staff and state and federal officials received a written disclosure statement prior to observations and interviews. Participants in the intervention consented to intervention observation and focus group participation prior to their participation in the study.

### Data Analysis

The Arizona and Sonora researchers employed a collaborative approach to data analysis; however, for pragmatic reasons each team took the lead on a specific data source. For initial analysis, the Sonora team, which worked extremely closely with the centers in conducting the intervention and collecting participant data, conducted initial analysis of the observation data with a focus on MSD delivery, GAM dynamics, the interaction between GAM facilitators and participants, and individual, family and work related factors expressed by facilitators or participants. The Arizona team took the lead in analyzing health center evaluation, stakeholder, and facilitator meetings to identify facilitators and barriers to implementation of the intervention in the context of the health centers, which then further informed the development of the decision maker interviews. The purpose of the initial stage of analysis was to develop a codebook based on the COACH tool as used by Blacklock et al. (40) that provided the lens of previous studies through which we were able to categorize contextual factors and identify issues relevant to implementation (Table 3). In this deductive analytical process, two members of the research team coded an initial sample of the relevant data set and established agreement regarding inclusion and exclusion criteria for each contextual factor or node. The Arizona or Sonora team then met to discuss coding definitions and identify sub-nodes. The binational team subsequently discussed findings across analyses and further refined the codebook. As a complex research study, aspects of the implementation study served as process evaluation for the cluster RCT of the MSD intervention (9, 13, 43).

**Table 3:** Meta Salud diabetes implementation study coding framework and themes.

**Contextual factors**	**Implementation questions**	**Emerging themes and illustrative quotes**
**MANAGEMENT**		
Availability, skills, motivation, and experience of local leadership	•Does management have power or leadership and resources to change aspects of the organization's structure and function to positively influence the impact of the intervention? (i.e., provide time for training; ensure staffing; support change; support teams)	•Many of the health centers had new directors due to a change in state government and there was frequent turnover of GAM facilitators, which meant a lack of continuity. •Health center directors varied in who and how many people they sent to MSD training (i.e., nurse, psychologist, community health worker, intern).“*The nurse in charge of the GAM should not change every 6 months. When a new nurse comes in [GAM], participants stop coming. The new person does not receive training. They just repeat topics without any continuity. You also need tro train the director and the head of nursing so they know what's being done.” (GAM facilitator, MSD feedback meeting)*
Performance monitoring and feedback	•What data is being monitored and how is it used for planning? •Is there a GAM annual work plan and is it being monitored? Who is responsible for it?	•Although the Ministry of Health required submission of an annual plan for the GAM, facilitators did not receive feedback. •The health centers submitted patient data from their registries to accredit their GAMs, but there was no process to verify the data.“*[We need to] change the mindset that things aren't going to work out. There are many support programs [for patients] but no one's paying attention to quality.” (GAM facilitator, MSD feedback meetings)*
Established institutional culture or behavioral norms that affect potential for change	•What is the view of health promotion? •How do providers perceive patients? How do staff communicate with patients?	•Health center directors felt that the intervention could be of great benefit to the staff and patients. Some health center directors were not familiar with GAM purpose or guidelines.“*The director trusted us completely to work on the [MSD] project, he gave us the freedom to do it, but we didn't sense that he was interested in taking part or being informed of the progress of the program. It ended up being ‘Do it however you want to do it, but do it’.” (GAM facilitator, MSD feedback meeting)*
**STAFFING**		
Availability of human resources and this is affected by staff turnover, pay, and incentives	•How are staff supported and incentivized? (i.e., trainings, travel support, clear job role, control over one's own work) What contributes to high turnover?	•GAM facilitators were supported by interns who are not available in the long run. •GAM facilitators and other staff did not receive regular health promotion training.“*Since there is very little staff [in the health center], each one of us has their own program, right? So there's a head of immunizaions, but if she has to do immunizations we have to help her out, so all of us help her out. And is if someone else [from another program] needs help we get it, but that's it. For example, I'm the head [of the GAM] right now, so I'm the one that's with [the group], but if at some point I can't do it or something, one of the girls can come and give them the talk.” (GAM facilitator, case study interview)*
Skills and knowledge	•Is there induction training for GAMS? •Ongoing supervision and in-service training? •How many people are trained in GAMs?	•Some GAM facilitators had never received health promotion training, particularly on chronic disease. •Facilitators were enthusiastic about in-person training.“*Our bosses are always coming and going to trainings in other places and don't necessarily replicate the training when they come back. Before they used to send other health center staff to the trainings but now they don't.” (GAM facilitator, MSD facilitator feedback meeting)*
Personal motivation and agency to affect change in the health center	•How are GAMS perceived by staff? •How are changes communicated and supervised?	•In some Centers, the GAM facilitator was assigned because no one wanted the responsibility. •In other Centers, the GAM facilitator had been in the position for years and had strong ties with participants, which encouraged motivation and agency.“*I can take the handbook and follow the instructions, but if I don't go the extra mile for the patient, so I can feel that I'm helping the patient, it's just going to be a regular work day. The attitude of the staff, that's what's going to determine if it works or not, if a program like this one is relevant or not.” (MSD assistant facilitator, case study interview)*
**LOCAL ENVIRONMENT**		
Patient and community factors that constrain health personnel such as language, cultural expectations, poverty	•What are the positive health seeking behaviors exhibited by patients and how can they be influenced? •How do patients perceive the GAMS?	•Patients faced economic challenges. •The patients had difficulty accessing care because of the health center's location and the lack of public transit. •The GAM participants were not interested in physical activity. •GAM participants wanted their family to participate in the GAM.“*Unfortunately [because of], our cultural roots we have to motivate the patients to participate, motivate them in any way. If they see something that motivates them they start to come to their groups. Particularly the facilitator, that's why the profile [of the GAM facilitator] that you mentioned is important. It has to be someone who's very dynamic and that above all else has a lot of communication with the patient and treats them as they should be treated.” (Health Center Director, case study interview)*
Lack of medication and material resources	•What are the constraints to care facing centers? GAMS facilitators? •How do we adapt to resources available?	•Staff at some health centers requested assistance obtaining diabetes medication. •Many GAMs had inadequate access to measurement equipment, including strips for glucometers.“*For GAM use, they share the baumanometer and scale they use for outpatient services, they do not have measuring tapes, and usually there are not enough test strips for the glucometer they use.” (Health center evaluation)*
Parallel and competing health system interventions from the state and federal level	•Are there any conflicting or concurrent policies, initiatives or programs that might positively or negatively impact the intervention? Overtax the staff? •What are the competing external factors on implementation of the program?	•Health promotion staff confront dueling priorities.“*[The GAM facilitator] mentioned that the Health Center was giving priority to other activities before the GAM, immunizations for example.” (MSD observation)*

## Results

Table 3 presents the initial results of the MSD implementation study that resulted from the progressive and iterative process of data collection and ongoing analysis. We modified definitions of the contextual factors based on the issues that emerged from the initial analysis. The team identified many of the implementation questions in the COACH tool based on initial health center evaluations and subsequent observations, but these too were refined through analysis and discussion. The implementation questions addressed in the framework further focused data analysis and development of sub-nodes for each contextual factor. The binational research team identified the emerging themes as characteristics of management, staffing, or the environment that we considered essential to the endeavor of MSD adoption, dissemination and scale up across the health system.

### Local Management

Within the sphere of management, support from the Director was associated with GAM/MSD implementation. We documented varying levels of support for health promotion, stemming from a curative rather than a preventive approach to clinical care. Directors demonstrated high levels of support by sending more people to the 3-day MSD training, as well as in prioritizing human and material resources for the GAM. The cultural and behavioral norms set by upper management also contributed to either interdisciplinary support from other staff for the GAM/MSD, or conversely to the GAM/MSD operating in isolation from clinical care. Supportive norms included an appreciation of the role of health promotion in health care, encouragement and rewards for teamwork, recognition of the value of the GAM for patients and the time commitment of the GAM facilitator. Across all the centers, lack of management support for the GAM was demonstrated and perpetuated by an overall laxity in evaluation; there was no evidence of verification of patient data submitted for GAM accreditation, and the required annual work plans were not being utilized for performance monitoring or feedback.

### Staffing

In the arena of staffing, GAM facilitators across the centers received no clear incentives for the work they do in the GAM. In some cases, those GAM facilitators had been assigned a duty that no one else wanted. Thus, positive attitudes toward facilitating MSD relied more on personal motivation and agency, with some GAM facilitators taking full responsibility for the MSD curriculum and others turning over the responsibility to student interns or other personnel. In addition, we documented a lack of standardized training and ongoing capacity building in chronic disease prevention and care for GAM facilitators and other health center staff. The MSD training thus operated as an incentive in most of the Centers, partly because the GAM facilitators saw it as an opportunity acquire new skills and network with their peers.

### Local Environment

Influences of the local environment on implementation were most evident in the inadequate supply of insulin, particularly in rural clinics, raising dilemmas regarding the role of health promotion when basic medical needs were not being met. Reasons for medication shortages were unclear; federal level decision makers speculated that the issue was distribution at the state level. State decision makers suggested it had to do with lack of timely programming at the health center level. In some cases, patients had to travel to access their medication, which was not always feasible. With respect to the socioeconomic environment, the MSD observations and case studies documented staff perceptions of patient economic and social characteristics, which led them to have low expectations that their patients would be able to engage in behavior change being addressed through MSD. Another challenge in the local environment was the perception that health center staff were over-extended by requirements to provide federally-sanctioned programs. These patient education and primary care programs addressed a range of health issues from infant development and adolescent sexuality to cancer prevention and infectious disease vector control, often on a competing timeline.

## Discussion

The delivery of evidence-based health promotion interventions within primary care has important implications for improving population health. While it can be challenging to merge implementation study processes into the more prescriptive and inflexible structure of an RCT, the integration of these two approaches potentially unveils design weaknesses or considerations that may ultimately undermine adoption of otherwise effective interventions. Qualitative and responsive methods allowed us to consider indicators relevant to both implementation and scale up, such as coverage, reach, adoption, site performance, and health outcomes (44, 45). These findings, in concert with conceptual frameworks and theories, provided a road map for future study of broader implementation and eventual scale up of the MSD intervention. In addition, the process of engaging the health system on center, district, jurisdictional, and state levels facilitated the identification of current systems of data collection and monitoring efforts relevant to evaluation of scale up, as well as gaps or inconsistencies in those efforts. The utilization of conceptual models that identified influential factors across the layers of the health system was essential in guiding the implementation study process.

The qualitative data had immediate implications for the clinical trial, as well as in informing future plans for scale up across the Mexican health system. The initial health center evaluations, for example, revealed space limitations and sensitized us to differences in how each center conducted the GAM. This evaluation then informed the elaboration of strategies of engagement for the centers that reflected the resources available. The application of qualitative methods in tandem with the RCT also allowed the design of one study to inform the other. Once in the field with the RCT, for example, we found that several of the conclusions we drew regarding the GAM infrastructure and internal support based on the health center evaluations were incomplete. We made the decision to observe all 13 MSD sessions at each intervention center to gain more comprehensive insight into how the varying context of each GAMs influenced the intervention. Additionally, we conducted observations of the GAMs in the control centers to gain further perspective on the usual practice of the GAMs. In being present each week to observe the MSD sessions, the study team identified gaps in data collection relevant to the models used to design the implementation study. For example, we became aware that the facilitators would benefit from additional training, but we were not clear what information or support they needed. We subsequently added the facilitator feedback meetings to document personal or environmental challenges to implementing MSD from the perspective of the facilitators. These additional activities were essential in detailing health center conditions vital to implementation, and identifying those actors within the centers who could potentially influence successful adoption by individual centers.

We applied this approach using iterative identification of appropriate theories and frameworks for qualitative data analysis. As we became increasingly familiar with the health system and the health center environment, we identified additional conceptual tools to guide our study. For example, based on the observation data, we identified the need for Normalization Process Theory (NPT) to investigate motivations of and relationships between center staff that were crucial to decisions to sustain the intervention after completion of the RCT. NPT informed design of subsequent research activities including feedback meetings, case studies, and interviews with state and federal decision makers.

A history of cooperation with stakeholders in the health system was critical to successfully conducting both the intervention and implementation studies. It is unlikely that we would have gained access to the health centers without our long-standing relationship with the Ministry of Health, which provided a foundation for open dialogue and discussion of the strengths and challenges of conducting an RCT. National concern regarding the current and projected health burden of diabetes in Mexico was a decisive factor in gaining support from the Sonoran Ministry of Health for the study. The extent to which the Ministry of Health facilitated commitment to the study among health center directors was rooted in the potential to identify an evidence-based program that could be applied to address chronic disease (9) in this context. Equally, evidence of effectiveness will be essential to build the political will for future translation efforts. Participatory methods, in particular using research liaisons who spent considerable time developing relationships with each center, assured the opportunity to capture multiple voices and sensitive issues within and across the clinics (28, 46). As with any health system, there were political considerations on every level, and our relationships with people in the Ministry of Health enhanced our awareness of these issues and helped us mitigate them.

### Limitations

Limitations of this study were related to challenges intrinsic to implementation science in that the research does not control for the environment, but seeks to describe and accommodate or adapt to it. The existing structure of the GAM introduced variation across centers, for example, since some health centers had regular meetings and existing participation that readily accommodated the weekly intervention, while others increased their meeting times and/or recruited new participants to the GAM to accommodate the study. Additionally, engagement of staff across the 12 health centers was inconsistent, with some centers being more interested in interacting with the research aspect of the intervention and others more focused on fulfilling their assignment to it. This may have resulted in the research not capturing contextual factors in those centers that were less engaged. The robust nature of the research methods that included capturing implementation from varying perspectives was an effort to address this issue.

### Future Directions

The diverse populations of Mexico require adaptation of MSD materials to local sociocultural settings, preferably by those most familiar with regional differences, particularly for indigenous population from over 60 different ethnic groups. MSD was designed to address the needs of those living in very vulnerable conditions, in this case, lower-income older adults with diabetes. Our analysis demonstrated that MSD alone cannot be successful without the provision of medications, especially insulin, and ongoing training for other medical personnel providing care to uninsured patients with diabetes. Additionally, our research was not designed to capture the social and environmental conditions of the population that make up the catchment areas for the health centers. Further research and implementation efforts need to address social conditions that will influence the effectiveness of this and other health promotion interventions, most importantly access to potable water, fresh foods at accessible prices and safe spaces that facilitate physical activity, particularly in the extreme climate conditions of Northern Mexico.

## Data Availability Statement

The datasets generated and analyzed during the current study are not available due to the fact that only the Co-PIs and Co-Investigators of the study have access to study data and study sites during the study period due to contractual agreements that limit such access for investigations. Following completion of the study data will be available upon request.

## Ethics Statement

The study protocol and consent process was approved by the University of Arizona Human Subjects Institutional Review Board. The protocol was also approved by the Research Bioethics Committee at the University of Sonora. The Collaborative Institutional Training Initiative (CITI) certified all research study staff on human subjects.

## Author Contributions

MI contributed to conceptualization of data collection, and data analysis and in writing the manuscript. CD oversaw study activities in Mexico, contributed to conceptualization of data collection and data analysis and participated in writing the manuscript. EC-V contributed to conceptualization of study design, data collection and data analysis and participated in writing the manuscript. MC-V contributed to conceptualization of study design, data collection and analysis. BA contributed to data collection and data analysis and participated in writing the manuscript. AO contributed to research design and data analysis. JZ contributed to conceptualization of study design, data collection and analysis and participated in writing the manuscript. CR oversaw all study activities, contributed to conceptualization of study design, data collection and analysis and participated in writing the manuscript.

### Conflict of Interest

The authors declare that the research was conducted in the absence of any commercial or financial relationships that could be construed as a potential conflict of interest.
