# Supplementary material for: The Meta Salud Diabetes Implementation Study: Qualitative Methods to Assess Integration of a Health Promotion Intervention Into Primary Care to Reduce CVD Risk Among an Underserved Population With Diabetes in Sonora, Mexico
Source: Front Public Health. 2019 Nov 15;7:347. doi: 10.3389/fpubh.2019.00347 (PMC6874016; doi:10.3389/fpubh.2019.00347)
Supplement: Supplementary file 1 [file Data_Sheet_1.PDF]

Interview Guide

For the case studies for Aim 2 for the project "Tools and practices to reduce cardiovascular disease and its complications in a diabetic population in Mexico"
Final Version: June 13, 2017

Center Director Questions

1. Why do you have a GAM in the health Center? (probe: describe the process/intent of establishing and certifying the GAM.)
2. How do you choose the GAM coordinator? Who selected this person?
   a. What are the responsibilities of the GAM coordinator?
   b. What is the profile of the ideal GAM coordinator?
3. How does it affect the health center that the GAM be accredited or not? What is the incentive for the center to accredit the GAM?
4. How have you incorporated the new GAM guidelines into the GAM? ("Support Group Strategy, Chronic Disease, Operation Guidelines")?
5. Does your health center have medical personnel to oversee chronic disease? Why or why not? What advantage or benefits does this mean for the GAM?
6. What type of training does the person in charge of facilitating the GAM need, as a person who is caring for people with diabetes?
7. What is your perception of Meta Salud Diabetes? What do you think of this approach with respect to health promotion and education for people with diabetes?
8. What challenges or barriers do you see in the success of a program like Meta Salud Diabetes?
   Probe: The following are themes that might be mentioned:
   a. Allusion to individual characteristics of the patient (difficult patient, neglect, ignorance, poverty, lack of family support).
   b. The relationship between doctors and people with diabetes
   c. Limitations in the quality of care at the health center
   d. Staff rotation and multitasking. Why does rotation occur?
   e. Lack of consistency / continuity in the programs of the Ministry of Health.
   f. Lack of involvement of medical, nursing and promotion staff.
   g. Lack of budget
   h. Lack of support to solve problems of infrastructure, logistics, etc. For example, the time of preparation of the sessions.
   i. i. Lack of materials such as alcohol, lancets, plasters, scale, glucometer.
9. What are the ideal conditions for a program like Meta Salud Diabetes to function well?
   a. How would you order these by priority?
   b. Would you consider this health center very close or very far from these ideal conditions? Why?
10. Has the involvement of Heath Center personnel changed since implementation of Meta Salud Diabetes?
11. Although the Meta Salud Diabetes sessions have ended, has your staff continued to use the materials? (who: GAM facilitator, doctor, nurse, intern?) (Probe: Do they continue with any of the interactive activities or physical activity routines?)
12. Have you participated in other projects or programs for people with diabetes? What projects and organizations? How did they manage them?
13. How do you prioritize use of the use of various health promotion programs from the Ministry of Health? If the programs are articulated, how are they articulated?

14. Do you think Meta Salud Diabetes could be integrated as a program of the Ministry of Health, that is, could it be part of a national health promotion strategy?
15. If you were in charge of scaling up a program like Meta Salud diabetes, how would you do it?

Questions for chronic disease providers;

1. What is the clinical protocol for a person when they are diagnosed with diabetes? Probe:
   a. Refer them to the nutritionist, psychologist, physical activity.
   b. What do you when the patient does not have their diabetes under control?
   c. At what point is a patient referred to UNEME?
2. Who is in charge of directing people to the GAM and who decides to send them?
3. What is your perception of Meta Salud Diabetes? What do you think of this approach with respect to health promotion and education for people with diabetes?
4. What challenges or barriers do you see in the success of a program like Meta Salud Diabetes?
   Probe: The following are themes that might be mentioned:
   a. Allusion to individual characteristics of the patient (difficult patient, neglect, ignorance, poverty, lack of family support).
   b. The relationship between doctors and people with diabetes
   c. Limitations in the quality of care at the health center
   d. Staff rotation and multitasking. Why does rotation occur?
   e. Lack of consistency / continuity in the programs of the Ministry of Health.
   f. Lack of involvement of medical, nursing and promotion staff.
   g. Lack of budget
   h. Lack of support to solve problems of infrastructure, logistics, etc. For example, the time of preparation of the sessions.
   i. i. Lack of materials such as alcohol, lancets, plasters, scale, glucometer.
5. What are the ideal conditions for a program like Meta Salud Diabetes to function well?
   a. How would you order these by priority?
   b. Would you consider this health center very close or very far from these ideal conditions? Why?
6. Do you think Meta Salud Diabetes could be integrated as a program of the Ministry of Health, that is, could it be part of a national health promotion strategy?
7. If you were in charge of scaling up a program like Meta Salud diabetes, how would you do it?

Questions for the GAM coordinator and other personnel responsable for facilitating the GAM:

1. Talk to us about the history of the GAM- when was it funded? How has it developed? How long have you been participating?
2. In general, what has been your experience implementing the Meta Salud Diabetes program?
3. What is your perception of the Meta Salud Diabetes program? What do you think of this approach with respect to health promotion and education for people with diabetes?
4. What kind of support do you receive in implementing Meta Salud Diabetes? (probe: What support did you receive from the Director? Was other personnel involved? Was their support of assistance from the District?
5. What were the principal barriers you encountered in implementing the Meta Salud

Diabetes Program in the GAM?

Probe: The following are themes that might be mentioned:

a. Rotation in staff duties (multitasking, lack of consistency in programs from the Ministry of Health) Why is there staff rotation?
b. Lack of involvement of medical, nursing and promotion staff.
c. Difficulty in understanding and teaching Meta Salud Diabetes session content.
d. Lack of support to solve problems of infrastructure, logistics, etc. For example, the time of preparation of the sessions. .

6. What are the ideal conditions for a program like Meta Salud Diabetes to function well?
   a. How would you order these by priority?
   b. Would you consider this health center very close or very far from these ideal conditions? Why?
7. What kind of training is needed to implement Meta Salud Diabetes, and in general to work in the prevention and control of chronic diseases such as diabetes and cardiovascular disease?
8. Now that you have had the experience of facilitating 13 Meta Salud Diabetes sessions, is there some aspect or content of the sessions that you would change or adapt? How and why?
9. Now that the Meta salud diabetes sessions are over and you continue working with the GAM do you think that the participants have more mastery over information related to hypertension and diabetes? How do you see this happening?
10. From your perspective, do you think that the GAM Works differently after doing Meta Salud Diabetes? Why yes or why no?
11. Has the involvement of the health center personnel in the GAM changed?
    a. Is there other personnel who ought to be involved in supporting or helping to faciliate the GAM and the Meta Salud Diabetes sessions?
    b. What do you see as the potential role of the interns?
12. Now that the University has stopped working with the GAM, do you think that Meta Salud Diabetes could be institutionalized in the GAM? Why?
13. Do you think Meta Salud Diabetes could be integrated as a program of the Ministry of Health, that is, could it be part of a national health promotion strategy?
14. If you were in charge of scaling up a program like Meta Salud diabetes, how would you do it?
15. How have you incorporated the new GAM guidelines into the GAM? (“Support Group Strategy, Chronic Disease, Operation Guidelines”)
